# Lessons in implementing open farm data for diverse stakeholders at Harper Adams Future Farm

**DOI:** 10.1093/af/vfag016

**Published:** 2026-05-27

**Authors:** Megan J Lewis, Steffan A R Newell, Alice Rogers, W Edwin Harris, Grace Milburn, Michael R F Lee

**Affiliations:** Centre for Agricultural Data Science, Harper Adams University, Newport, TF10 8NB, UK; Future Farm, Harper Adams University, Newport, TF10 8NB, UK; Future Farm, Harper Adams University, Newport, TF10 8NB, UK; Centre for Agricultural Data Science, Harper Adams University, Newport, TF10 8NB, UK; Future Farm, Harper Adams University, Newport, TF10 8NB, UK; Future Farm, Harper Adams University, Newport, TF10 8NB, UK

**Keywords:** decision support, digital farm systems, FAIR data, farm data, open data

## Abstract

**Graphical Abstract:**
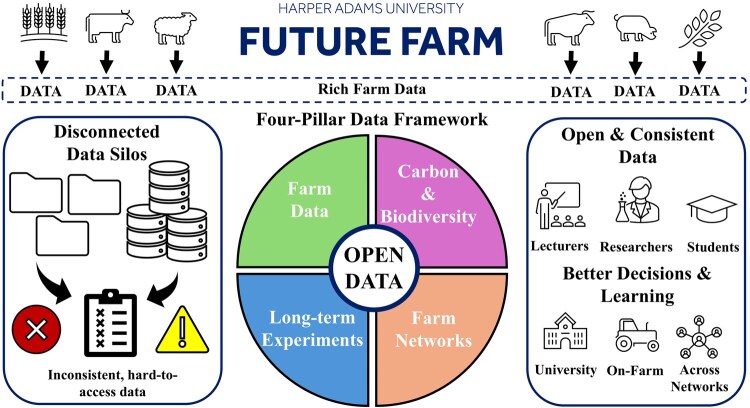

Implications

- Wider sharing of agricultural data is essential for innovation and progress in the agricultural sector. However, most data remain confined to limited stakeholder groups via third-party systems.
- Alongside concerns of data ownership and privacy, inconsistent data formats, limited platform interoperability, and the curation of high-quality datasets are shared challenges across commercial, research and educational farms.
- Following its “*Four Pillars of Farm Data*,” Harper Adams Future Farm is working toward a more collaborative and innovative future for agricultural data by actively promoting and integrating open data principles into everyday processes.
- Research and education farms equipped with multidisciplinary support teams are arguably better positioned to curate practical, high-value datasets.
  Education and research farms should aim to demonstrate practical open data systems, acting as key knowledge hubs to support the next generation with the skills required for wider adoption in an increasingly data-driven agricultural landscape.

## Introduction

Agriculture is a diverse sector comprising of farming businesses ranging from small family-run farms through to corporate, large-scale systems. Beyond this, farm businesses further diverge based on their system focus (e.g., arable and broadacre, livestock-specific, organic, etc.). Yet despite these vast differences, the entire agricultural sector faces the same challenge of increasing productivity while maintaining sustainability and economic viability (Giller et al., 2021). While some farmers may choose to partake in environmental schemes or diversify their businesses with alternative income streams (e.g., glamping sites, farm shops, etc.), another approach focuses on the adoption of technology and rigorous collection of farm data for evidence-based decision-making.

Agricultural data is often complex and heterogeneous, collated from a range of disparate sources and collected by a variety of methods (e.g., Janssen et al., 2017; Top et al., 2022; Falcão et al., 2023). Consequently, a desire for data-driven, evidence-based decisions has led to more automated data collection methods using technological developments like the Internet of Things (IoT), remote sensing and cloud computing (Foresight, 2011; Fountas et al., 2020; Uyar et al., 2024). Sensors, automated recording systems combined with data platforms can collect data continuously and with greater precision than traditional manual recording methods (Basir et al., 2024). This has the potential to free up time for farmers who are often time-poor, while also increasing the volume, frequency, and consistency of data captured. When used appropriately, current data and future data generated from farms have the potential to improve economic and environmental performance at the farm-level but could also lead to benefits at a wider sector-level (Elijah et al., 2018; Kayad et al., 2022).

The progress toward reaching commercial farm business goals through data can be further enhanced by sharing relevant data with key industry stakeholders (e.g., agronomists, nutritionists, veterinarians, etc.). In research and educational farms, the list of stakeholders expands even further (e.g., researchers, students, educators, etc.), where sharing farm data can play a pivotal role in advancing agricultural innovations, especially when combined with wider collaboration with the commercial sector (Rozenstein et al., 2024). However, within the wider agricultural sector, the sharing of data is often limited to individuals and small groups of these stakeholders via proprietary digital platforms that require access permissions (Kalmar et al., 2022). While this can lead to benefits for individual farms, it can limit the potential for new discoveries that could benefit the wider sector. As such, achieving wider accessibility to agricultural data is vital to maintaining innovative progress within agriculture.

The management of data for open accessibility by stakeholders is a challenge across many sectors. Several frameworks and models have stemmed from the desire to make data more accessible, including what is commonly referred to as “open data.” Open data itself has no single definition across sectors, but is generally accepted as providing data in a manner that allows it to be freely accessed and used by anyone (Jati et al., 2022). Another more defined framework for sharing data is the FAIR data concept, which defines four principles to increase the value of data assets: data should be Findable, Accessible, Interoperable, and Reusable (Wilkinson et al., 2016). The use of FAIR principles in agriculture has been limited perhaps due to a lack of time or skills to appropriately format and catalog data (Falcão et al., 2023; Kumar et al., 2024; Rozenstein et al., 2024; Kutha Krisnawijaya et al., 2025), but data ownership and privacy are also key concerns. The question of “who” will have access to data and “how” they may utilize it remains a large barrier within the commercial sector as well as the time and skills to appropriately format and catalog data (Falcão et al., 2023; Kumar et al., 2024; Rozenstein et al., 2024). Often farmers may be reluctant to share data if the purpose of its use is unclear or if there is uncertainty around how it may be interpreted, distributed, or monetized by third parties (Zhang et al., 2021).

While the sharing of agricultural data can be challenging, some farms may be better positioned to share data. For example, farms associated with research institutions are likely to be motivated to share data as part of the research and publication process (Umbach, 2024), while sharing data in educational farms can improve collaboration and allow for more authentic learning and research impact (Kjelvik and Schultheis, 2019). Farms within networking systems can also benefit from additional support and knowledge exchange that allows them to share their data more widely. For example, the Global Farm Platform (GFP) employs a “hub and spoke” network structure where the “research and educational” farms act as the hubs and the commercial farms the spokes, sharing data between them (Rivero et al., 2026). In the context of the GFP, sharing farm data within the network is central to improving farm practices, such as optimizing nutrient management and supporting environmental protection. Furthermore, data flow is two-way, with innovations often first trialed on commercial farms before being shared across the network.

In this article, we describe the journey that Harper Adams Future Farm (HAFF) and the agricultural data team have been on to reach open and accessible data. We highlight our current farm setup, our foundational motivations, and what we have achieved so far. Finally, we discuss our plans and directions for the future with an emphasis on what is needed to reach FAIR data at HAFF and our role in the wider Global Farm Platform and National Farm networks to advance sustainable farming practice.

### Harper Adams Future Farm

HAFF is a working agricultural enterprise, a research demonstrator site, and a platform for agriculture education led by the principles of commercial best practice. It forms an integral part of teaching, research, and external collaboration at Harper Adams University in Shropshire, United Kingdom. HAFF provides real-world context for students, researchers, and industry partners across a range of animal- and land-based disciplines. The farm is regularly used for student teaching and demonstrations, and frequently hosts visitors and collaborators, meaning that decisions made on the farm often have implications beyond commercial performance alone. HAFF is a member of the Sustainable Farm Networks in the United Kingdom, connecting ca. 2,000 commercial farmers to realize more sustainable farming practices. Internationally, the farm is connected via its membership of the Global Farm Platform, a collaboration of 28 global research farms focusing on sustainable ruminant livestock systems. Both these networks position HAFF as a “lighthouse farm” to lead other farms toward more sustainable practice. Both positive and negative learnings of trial work (e.g., stripping phosphorus and nitrogen from slurry) are shared with the networks, which facilitates discussion and interpretation for wider adoption and improvement across a broad spectrum of stakeholders (e.g., researchers, farmers, extension agents).

HAFF is structured as a series of linked livestock- and land-based enterprises operating within clear physical and managerial boundaries. The site extends to around 500 hectares and is managed as a commercial mixed farm. All major livestock enterprises (dairy, beef, sheep, and pigs) support active research, while also contributing to day-to-day farm outputs. Grassland and arable land are used in combination to support the livestock enterprises, with most forage grown on-farm and rotations planned around both production requirements and teaching needs. Manure from the liquid slurry system is returned to the fields, creating a circular nutrient cycle that links animal management with soil and crop decisions.

### HAFF data management challenge

Data generated across HAFF reflects the diversity of its enterprises. Information is captured through a combination of automated digital systems, manually entered electronic records and paper-based documentation. These datasets include animal health and performance records, production metrics, environmental indicators, and operational information, and are collected using disconnected systems (“data silos”) that have developed independently over time.

Managing data on a university-operated research and education farm led by the principles of commercial best practice presents additional challenges compared with typical commercial farms. Alongside normal operational needs, the same datasets must also support research, teaching, and external reporting, each with different expectations about granularity, timeliness, and accessibility. These issues mirror findings from wider agricultural data research, where fragmented systems and multiple stakeholder demands complicate reuse and governance (Wolfert et al., 2017; Chamorro-Padial et al., 2025).

At HAFF, routine data requests highlight these tensions. Managers need current figures for operational decision-making, researchers require detailed historical datasets in specific formats, and students and lecturers seek data suitable for project work and teaching, respectively. Much of this information is stored in separate software systems, each using different structures, permissions, and conventions. Every request requires manual extraction, cleaning, and reconciliation before it can be shared because these data silos are unconnected and different users can end up working from different versions of the same dataset.

These recurring issues made it clear that the problem was not simply a lack of digital tools. What was missing was a consistent way to organize, govern, and share data across the whole farm, wider university community and ultimately our national and international network partners. We, therefore, turned to approaches developed within research to improve the findability and re-use of data, and were particularly influenced by the FAIR principles (Wilkinson et al., 2016). In practice, we acknowledged that aligning with true FAIR standards would be a long-term challenge. Therefore, we decided to start with a modification of FAIR, which for us meant aiming for data that could be located easily, traced to its source, and reused with minimal reformatting, rather than making all data openly available to everyone, although this may ultimately be an outcome.

### The four pillars of data

**Figure 1.**
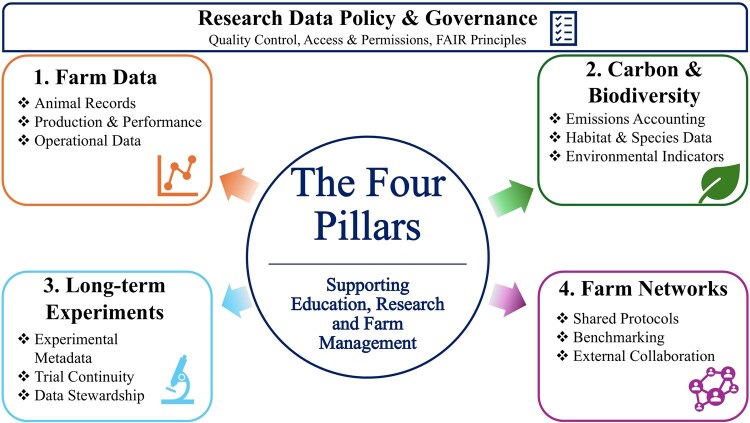
The Harper Adams Future Farm “Four Pillars of Data” framework. The pillars (farm data, carbon and biodiversity, long-term experiments, and farm networks) work together to support education, research, and farm management, under an overarching research data policy and governance structure.

In 2022, HAFF implemented the “Four Pillars of Data” as a theme to guide our data management journey (Figure 1). The pillars focused on 1) Farm production data; 2) Carbon and biodiversity; 3) Long-term experiments (unique experimental field trials investigating specific management approaches and innovations); and 4) Farm networks (e.g., Sustainable Farm Networks and Global Farm Platform), with our overarching aim to improve data stewardship across all pillars.

To support the four-pillar structure, we developed a central data portal page on the Future Farm website (https://www.futurefarm.zone/1290/data-portal/) to provide a single, clearly signposted location where any stakeholders using farm data can identify the relevant systems and resources.

#### Farm data—Pillar 1

A priority in our data journey was to establish a central platform for core farm data relating to livestock, cropping, inputs, performance measures, and operations. Historically, data were held across a mix of spreadsheets, individual software packages such as Omnia Hub (Hutchinsons) for cropping records and UNIFORM (UNIFORM-Agri) for dairy management, and paper files. This made it challenging for farm staff, researchers, and students to locate reliable, consistent data without considerable manual effort.

**Figure 2.**
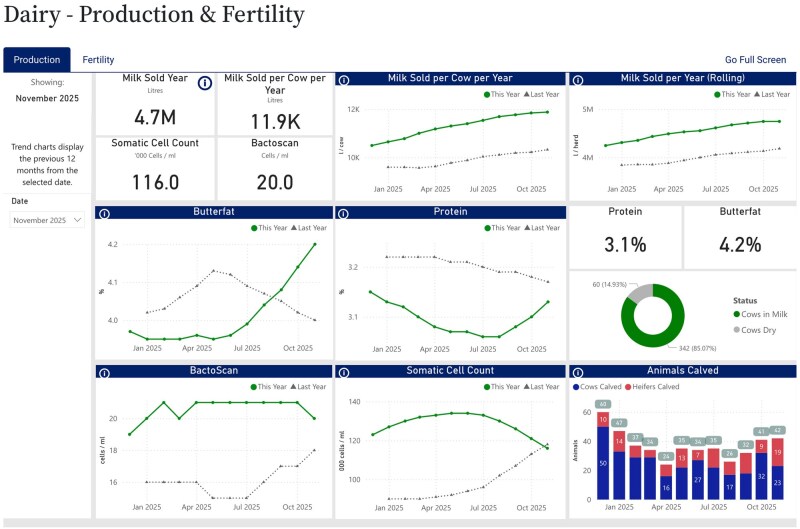
FarmMetrics dairy production and fertility insight dashboard (Map of Agriculture Group Ltd 2026) . The dashboard presents key performance indicators (e.g., milk yield and composition), consolidating data from multiple dairy data systems. Users can view trends over time and compare current performance with previous periods.

**Figure 3.**
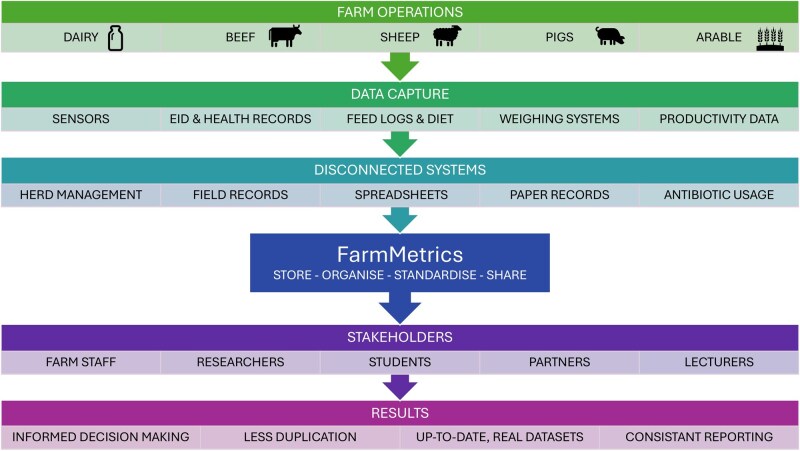
The diagram illustrates how data generated through routine farm operations could increasingly be captured, organized, and brought together through FarmMetrics, reducing fragmentation across data silos. The aim is for the portal to provide a single point where datasets are standardized and shared with different stakeholder groups, supporting better decision-making and more consistent reporting. The figure represents the direction of development rather than the current state of full integration.

To address this, we began working with Map of Agriculture Group Ltd (2026), a company specializing in integrating farm data from multiple sources to develop a central data platform, FarmMetrics. This platform brings data from the data silos into a single location, where it can be viewed, downloaded, and analyzed. The portal includes interactive insight dashboards (Figure 2) that visualize commonly requested information and key performance indicators. This enables users to see summaries and trends in one place rather than creating reports manually. In addition to these dashboard functions, FarmMetrics also incorporates a “what-if” greenhouse gas (GHG) modeling tool, which uses the underlying data to estimate emissions at the enterprise level. Users can explore how changes in management or inputs may affect GHG emission outputs. Model results can be accessed and compared to support discussion and planning. Users are also able to download underlying datasets, allowing raw or minimally processed data to be accessed for teaching, student projects, and research where more detailed analysis is required. Our aim is that this shared platform will reduce duplicated effort, ensure greater consistency in the data used across the university, and provide clearer, visual ways of supporting management, teaching, and research discussions. Our current plans for improving integration and access are summarized in Figure 3. This model represents the trajectory we are working toward, rather than a fully implemented system.

Although FarmMetrics represents an important step toward a more coherent data environment, it has highlighted several limitations. Not all legacy systems can be easily connected or kept up to date without manual intervention, and some enterprises are better represented than others. For example, the dairy unit produces high-resolution data from multiple automated systems, whereas arable and environmental records are more sporadic and currently rely on manual entry. Historical datasets often need cleaning and standardizing before they can be incorporated, which can be time-consuming and reveal gaps that were not apparent when the data were used only for day-to-day management. These issues have reinforced that building a central platform is only part of the solution. Sustained progress depends on improving data quality, agreeing on shared processes, and supporting staff and students in using the system confidently.

Looking ahead, FarmMetrics has been designed to grow alongside the farm. As additional systems are developed or upgraded, more data will be connected directly through automated integrations, reducing the need for manual uploads and improving consistency. We also anticipate opportunities to introduce more advanced analytical tools over time, including the use of AI-driven tools to support pattern recognition, reporting, and decision-making.

#### Carbon and biodiversity—Pillar 2

Alongside improving core farm records, we recognized the need for a clear and consistent approach to understand HAFF’s GHG footprint, particularly in a form that allows meaningful comparison between years. We, therefore, adopted Agrecalc, an independent farm carbon calculator widely used to quantify and benchmark GHG emissions across mixed farming systems (Agrecalc, 2026). The selection of Agrecalc was guided by industry principles for carbon accounting tools, including transparency of assumptions, repeatability, and suitability for commercial farm decision-making (AHDB, 2026). Using the same platform each year provides a consistent basis for comparing whole-farm and enterprise-level emissions over time.

**Figure 4.**
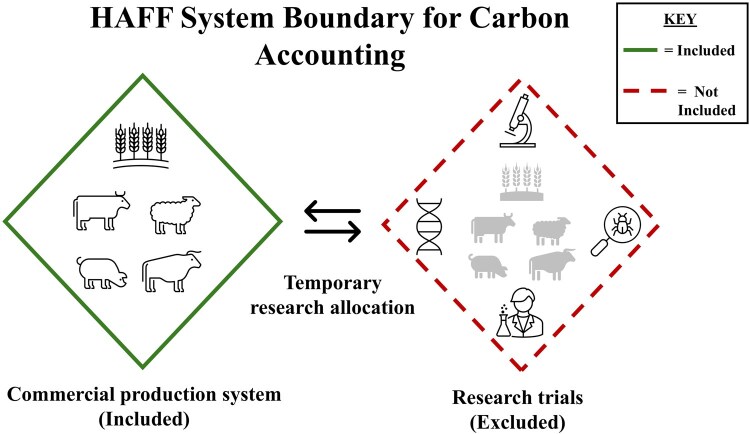
System boundary used for carbon accounting at Harper Adams Future Farm (HAFF). Commercial production is included in carbon accounting, while animals and land in research trials are excluded.

A key consideration in applying carbon accounting on a research-active commercial farm has been the definition of clear system boundaries. For carbon reporting purposes, HAFF is represented as a stable production system, with enterprise boundaries defined consistently from year to year. Where animals are temporarily removed from enterprises for research trials, adjustments are made so that emissions reporting reflects the underlying commercial system rather than the effects of experimental design (Figure 4). This approach allows for like-for-like comparison of enterprises, while still accommodating research activity on the farm.

Agrecalc reports are now available for the period 2022–2025. These draw on routinely recorded data, including livestock numbers, feed and fertilizer inputs, and crop outputs, to estimate emissions at both enterprise and whole-farm level. Having multiple years of results has improved understanding of where emissions arise across enterprises and supports internal discussion of mitigation options and can also be shared within our networks.

This process has also highlighted the importance of data integration and governance. Producing timely carbon reports depends on assembling information from multiple, disconnected data silos, which can introduce delays. In addition, while Agrecalc provides some limited benchmarking against comparable farms, these comparisons should be interpreted with caution. Carbon accounting is not yet a regulatory requirement, meaning benchmark datasets are often drawn from a relatively small group of more engaged or progressive farms, which can skew comparisons. As with other areas of farm data, meaningful carbon reporting, therefore, depends not only on the calculator used, but on consistent systems definitions, data quality, and clear governance processes.

Biodiversity is an important component of agricultural sustainability and is a key concern globally (Prestre, 2017). As such, we aimed to develop a baseline that could be used to monitor biodiversity and measure the impact of environmental management practices across HAFF and the wider University estate over time. We were also keen to ensure that the data would be available to staff, students, and collaborators to enhance teaching and research.

During initial investigations of our historical biodiversity data, we observed that the data had been collected somewhat sporadically across time and space. The types of habitat or taxon groups recorded tended to be driven by the interests and expertise of staff and students, leading to gaps in taxon data over time. Occasionally, data were also collected by external consultants as part of planning procedures. Due to the diversity of data collectors and methods, the data were stored in disparate locations, under different file formats, and often lacked metadata. It was clear that we would require more targeted planning and consultation with our staff to reach FAIR data standards. As a result, we initially focused on bringing biodiversity data into a single location via a bespoke dashboard developed by our data team. We started with our most intact biodiversity dataset available, namely our Breeding Bird Survey data.

**Figure 5.**
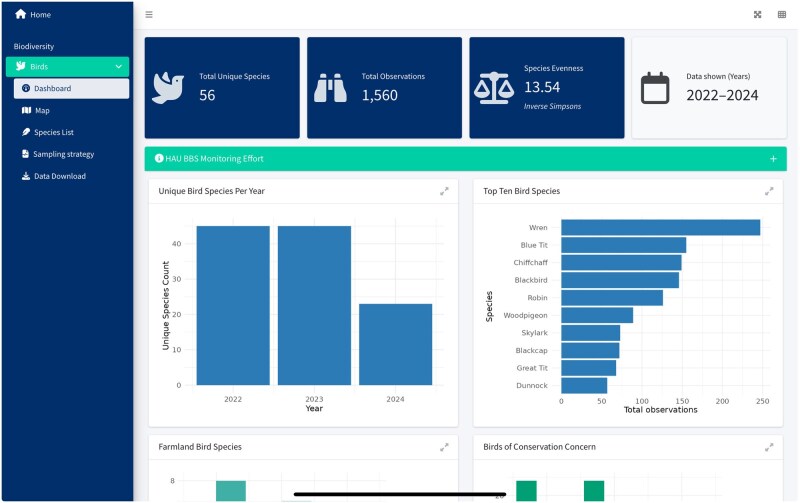
A snapshot of the Harper Adams biodiversity dashboard illustrating an overview of bird biodiversity based on the Harper Adams Breeding Bird Survey between 2022 and 2024. The dashboard incorporates key metrics (e.g., total unique species, total observations, and species evenness) and graphs showing unique bird species recorded each year and the overall top 10 species recorded. The dashboard shown reflects the functionality at the time of writing.

Staff members within the Agriculture and Environment Department have performed an adapted Breeding Bird Survey each year since 2022, collecting information on observers, weather, time, date and location in addition to the species information. Our initial preliminary dashboard focused on key overview parameters like total species recorded and a breakdown of key species groups (Figure 5). We also included an interactive map to display transect lines and individual observation records, the survey methodology, and a data download feature.

The dashboard is a continuously developing system that we plan to add to over time as we sort through the remaining historical data and as new data is collected. We foresee the need for the dashboard to be adaptable as we integrate different data from both manual and automated technological sources, as we receive feedback from users and as we actively review our goals.

#### Long-term experiments—Pillar 3

Long-term experiments have the potential to provide valuable novel information in domains like agriculture and ecology, especially when combined with external data (Perryman et al., 2018). However, long-term experiments are often complex with many potential sources of variation, even in the core data collected year on year. This variation, alongside the collection of extra data to answer additional specific research questions, adds further challenges in adhering to FAIR data principles (Ostler et al., 2023).

Harper Adams Future Farm currently has four long-term experiments of varying maturity, including:

- Traffic and Tillage: Established in 2011, and our longest running project, investigating the interaction between traffic and tillage on soils, crops, and production economics (Smith et al., 2013)
- Hands Free Farm: Exploring the automation and autonomy of crop production, including fleet management, swarm logistics, and economics (Franklin et al., 2025)
- Regenerative versus Conventional Agriculture: Investigating the impact of regenerative practices using a system-level design to determine optimal drilling date and weed control measures.
- Paludiculture Innovation Project: documenting the transition of arable farmland on valuable peat soils to a paludiculture system while developing rewetting techniques and evaluating the impacts on environmental factors (e.g., carbon and water), biodiversity, and identifying suitable crop options.

Historically, these projects have been managed as independent research projects and data management, including intellectual property considerations, has been confined to individual researcher teams. We have begun working with the research teams of these respective projects to identify and record metadata by adopting the Global Long Term agricultural Experiments Network (GLTEN) schema (https://github.com/GLTEN/GLTEN-Schema). This initial phase will allow the data team to understand the history of these experiments and identify potential datasets for collation and will also ensure that the data is potentially valuable to others (e.g., for meta-analysis, for students, etc.). We plan to continue to work closely with the research teams to collate data to establish useful assets with appropriate metadata and identify the need for any access restrictions in preparation for publishing the datasets. As these projects are ongoing, our efforts are a continuing process with plans to meet and maintain FAIR standards for our long-term experiment data.

#### Farm networks—Pillar 4

Collaboration and knowledge exchange are motivating factors for the sharing of agricultural data, and farm networks can play a key role in facilitating this collaboration. HAFF is a member of two connected networks based on the hub and spoke mechanism (Rivero et al., 2026), with HAFF acting as the “hub” and the national network of commercial farms acting as the “spoke.” The Sustainable Farm Network is a free-to-join, pre-competitive, system-agnostic forum working with existing knowledge exchange platforms to amplify successes and highlight challenges. The network was formed by the School of Sustainable Food and Farming at Harper Adams University (https://www.schoolofsustainablefoodandfarming.org/953/sustainable-farm-networks/). There are currently 37 member networks, representing over 2,000 farm demonstrators across hundreds of thousands of hectares spanning all United Kingdom sectors, systems and nations. The network collaborates to implement and advance sustainable farming practices, principles or technologies with the intention of disseminating the resulting collated findings, experiences or information. Internationally, HAFF is a “hub” farm within the Global Farm Platform (www.globalfarmplatform.org), a global network of model farms established to develop transformational solutions to challenges confronting sustainable ruminant production and promote their adoption. This multidisciplinary international network provides a unique combination of research and practice for diverse ruminant production systems in a wide range of cultural, socioeconomic, and climatic zones (Eisler et al., 2014; Rivero et al., 2021). The aim of Pillar 4 is to connect farm data platforms, using common tools and lexicon, across these networks to significantly enhance understanding toward more sustainable agricultural practices both nationally and internationally.

### Looking to the future

We still have developments to make and challenges to overcome, but our experience shows that moving toward open or FAIR farm data is not a quick technical fix, but rather an ongoing process. In the next year, we aim to integrate more systems from across the farm into Pillar 1 and expand the available biodiversity and long-term experiment datasets. We also plan on launching our university-wide research data policy to promote best practice and FAIR guidelines for data stewardship where appropriate. To support this, we have established a Harper Adams Dataverse to improve findability and accessibility with Digital Object Identifiers (DOI) and high-quality metadata. Both the research data policy and the use of the Dataverse will allow us to ensure fair attribution of contributions to collecting and curating datasets.

As we have previously highlighted, the wider sharing of agricultural data is essential for continued innovation and progress within the sector (Rozenstein et al., 2024). While our efforts have so far focused on improving data availability and accessibility internally at HAFF, we recognize that research and education farms also have an opportunity to demonstrate practical approaches to more open farm data systems. By trialing data platforms, governance structures, and workflows within a working commercial environment, HAFF and similar sites can act as testing grounds for approaches that may later be adopted more widely across the agricultural sector. We also acknowledge that university farms may have advantages not always available to commercial businesses. Research funding, student-led projects, and access to multidisciplinary expertise across the university can provide additional capacity to experiment with new technologies and approaches to data management. As such, the increased “person” capacity can effectively increase the time available for activities that are often difficult to prioritize within the operational constraints of a commercial farm such as data curation, integration, and analysis. With these additional advantages, the data and knowledge generated within university-associated farms must be shared beyond the university setting so that lessons learned can benefit the wider agricultural sector.

Furthermore, through our higher education activities, we are well-positioned to influence and contribute to the development of future farming professionals. Integrating farm data management, digital tools, and the principles of FAIR data into our curriculum ensures that the next generation of farmers, advisors, and researchers are well equipped to work within an increasingly data-driven landscape. As a university, we often provide short courses for continuing professional development across a variety of subject areas. In this sense, we have the potential to reach beyond the university cohorts and support current farmers and industry professionals in developing data literacy and technical skills. By collaborating with partner networks to reach these audiences, we have the potential to develop valuable resources that meet the explicit needs of current agricultural professionals.

Research and education institutions often have avenues to shape the wider conversation around agricultural data governance which is vital to tackling concerns of data privacy and ownership (Zhang et al., 2021). At HAFF, through collaboration with our School of Sustainable Food and Farming, we are well-positioned to lobby for greater support for farmers and to develop sector-wide guidelines and best practices for open farm data. This includes clearer frameworks around data ownership, privacy, appropriate data use and safeguards to ensure farmers retain confidence and protection when sharing their data. Strengthening these frameworks could help build confidence among farmers and industry stakeholders and ultimately support a more open and collaborative approach to agricultural data use.

## Conclusion

A commercially active farm that also supports teaching and research generates large and complex datasets, and bringing these together in a usable way is challenging. Our four-pillar framework has helped us identify what we need to reduce barriers to open farm data within our own system. However, many of the challenges we faced are also seen across the wider industry and university farms, like ours, can play a role in testing and demonstrating practical approaches to open farm data.

There is still work to do, particularly around older datasets, automation, long-term governance, and open accessibility of data, but we remain positive. We have unified many data systems and are consciously asking how we can continue to improve data access and stewardship to reach our long-term goal of open and FAIR farm data. We are continuously thinking about how we can embed awareness of open and FAIR principles into everyday practice to ultimately support farm management, research, and teaching and promote more open collaboration across the farming community nationally and internationally.

## Author Biographies

**Dr Megan J. Lewis** is a postdoctoral research assistant in farm data at Harper Adams University. She earned her master’s in Entomology from Harper Adams University and a PhD in Animal Sciences from the University of Western Australia. Previous research has focused on the impacts of veterinary medicines used in livestock husbandry on nontarget organisms such as dung beetles and how more sustainable targeted used could benefit important invertebrate populations. Currently, her work combines data science with agricultural biodiversity to improve collection, collation, and storage of biodiversity data for use in education and research.

**Steffan A.R. Newell** is a Data Officer at Harper Adams University’s Future Farm, where he is responsible for the development, management, and application of farm systems and farm data to support research, teaching, and sustainable farm management. His work focuses on improving data quality, interoperability, and the practical use of on-farm data to inform decision-making across commercial and research farm environments. He holds an integrated master’s degree in environmental science (MEnvSci) from Bangor University, and his academic research includes a dissertation examining approaches to lowering carbon emissions through the reuse of materials, reflecting a broader interest in sustainability, and resource efficiency. Prior to his current role, he worked in clinical trial data management at the University of Manchester and held comparable data management positions within the NHS, supporting the governance and analysis of complex datasets in regulated environments.

**Alice Rogers** is a Data Officer for Future Farm at Harper Adams University. She achieved her Masters in Sustainable and Efficient Food Production at Aberystwyth University after studying the potential effect dairy cow breeds could have on the concentration of milk urea and the relationship between milk urea and urea output in urine. She has previously worked as a Trials Officer assisting with research and commercial trials at the Crop and Environmental Research Centre at Harper Adams University.

**Dr W. Edwin Harris,** FRSS, Reader in Statistics and Director of the Agriculture Data Science Research Centre, is a data scientist with 20 years of experience in experimental design, data analysis, research, and consultancy across academia and the private sector, with expertise in agriculture, ecology, and conservation. He is experienced developing complex data-analytic solutions and communicating results to diverse stakeholders. He manages outreach and knowledge exchange between researchers, government, and the private sector, and leads the development of ag-tech research addressing agricultural and environmental impacts. He oversees statistical support services, capacity building for research staff and postgraduate students, and consultancy activities in both academia and industry. His current research focuses on adapting foundational artificial intelligence methods to agri-tech and ecological challenges.

**Dr Grace Milburn** is interim Director of the Future Farm at Harper Adams University. She earnt her PhD in soil chemistry studying the effect of granulated biosolid applications as a form of fertilizer. She previously managed the Crop and Environmental Research Centre at the same university, conducting teaching, research, and commercial trials. This work focused on fertilizer, agrochemical, and varietal inputs.

**Professor Michael Lee,** FRSA, FRSB, FRASE, ARAgS, is an expert in sustainable livestock systems, defining their role in securing global food security at the same time as protecting environmental health (Livestock’s role in human and planetary health). He graduated with first class honors in Animal Science from University of Wales, Aberystwyth in 1997, and gained a PhD in ruminant nutrition (protein and energy metabolism) from the University of Aberdeen in 2001 followed by Postgraduate certificate for teaching in higher education from Aberystwyth University in 2012. In November 2020, Michael moved from Rothamsted Research and the University of Bristol (where he held a joint position) to his current position as Deputy Vice Chancellor of Harper Adams University, England’s Premier specialist agriculture and land use University.
